# Transcriptome analysis reveals gene responses to herbicide, tribenuron methyl, in *Brassica napus* L. during seed germination

**DOI:** 10.1186/s12864-021-07614-1

**Published:** 2021-04-23

**Authors:** Liuyan Wang, Ruili Wang, Wei Lei, Jiayi Wu, Chenyang Li, Hongsong Shi, Lijiao Meng, Fang Yuan, Qingyuan Zhou, Cui Cui

**Affiliations:** College of Agronomy and Biotechnology, Southwest University, Chongqing, 400716 China

**Keywords:** Tribenuron methyl, *Brassica napus* L., Seed germination, Transcriptome, Physiology

## Abstract

**Background:**

Tribenuron methyl (TBM) is an herbicide that inhibits sulfonylurea acetolactate synthase (ALS) and is one of the most widely used broad-leaved herbicides for crop production. However, soil residues or drifting of the herbicide spray might affect the germination and growth of rapeseed, *Brassica napus*, so it is imperative to understand the response mechanism of rape to TBM during germination. The aim of this study was to use transcriptome analysis to reveal the gene responses in herbicide-tolerant rapeseed to TBM stress during seed germination.

**Results:**

2414, 2286, and 1068 differentially expressed genes (DEGs) were identified in TBM-treated resistant vs sensitive lines, treated vs. control sensitive lines, treated vs. control resistant lines, respectively. GO analysis showed that most DEGs were annotated to the oxidation-reduction pathways and catalytic activity. KEGG enrichment was mainly involved in plant-pathogen interactions, α-linolenic acid metabolism, glucosinolate biosynthesis, and phenylpropanoid biosynthesis. Based on GO and KEGG enrichment, a total of 137 target genes were identified, including genes involved in biotransferase activity, response to antioxidant stress and lipid metabolism. Biotransferase genes, *CYP450, ABC* and *GST*, detoxify herbicide molecules through physical or biochemical processes. Antioxidant genes, *RBOH, WRKY, CDPK, MAPK, CAT,* and *POD* regulate plant tolerance by transmitting ROS signals and triggering antioxidant enzyme expression. Lipid-related genes and hormone-related genes were also found, such as *LOX3, ADH1, JAZ6, BIN2* and *ERF*, and they also played an important role in herbicide resistance.

**Conclusions:**

This study provides insights for selecting TBM-tolerant rapeseed germplasm and exploring the molecular mechanism of TBM tolerance during germination.

**Supplementary Information:**

The online version contains supplementary material available at 10.1186/s12864-021-07614-1.

## Background

Weed competition is an important limiting factor affecting crop yield [1]. Tribenuron methyl (TBM) is an herbicide that acts by inhibiting sulfonylurea acetolactate synthase (ALS), which reduces isoleucine, leucine and valine biosynthesis [2]. Rapeseed is a broad-leaved crop and therefore more sensitive to TBM, so TBM is rarely used directly for weed control in rapeseed production. However, planting methods such as rotation or intercropping can leave TBM residues in soil and herbicide spray can drift onto other areas [2, 3], which might cause physiological and biochemical changes that inhibit germination or reduce seedling quality and crop growth and development.

The biological stress response to adverse environmental factors is essentially the result of differential gene expression. Gene chip and high-throughput sequencing technologies have played a major part in the identification of genes related to the stress response at the whole genome level. The complete sequencing of the rapeseed genome also provided valuable molecular resources for studying the mechanism of stress resistance [4]. RNA-seq analysis can quickly identify differently expressed genes (DEGs) between different samples. Up to now, RNA-seq has facilitated the identification of DEGs in rapeseed under abiotic stresses such as drought [5], freezing [6] and salinity [7], as well as some candidate genes related to sclerotinia [8], seed aging [9], seed coat color [10], and flowering time [11]. Some studies on herbicide stress to plants were also carried out using RNA-seq. For example, after treatment with TBM, single nucleotide polymorphisms (SNPs) [12] and non-target-site resistance (NTSR)-related genes such as for glutathiones, peroxidases, oxidases, hydrolases, and transporter proteins were identified in *Myosoton aquaticum* L. (water chickweed) [13], short-awn foxtail [14], grain sorghum [12] and rye grass [15]. Application of TBM affected root and above-ground growth of cornflower [16], and reduced the biomass of foxtail millet [17]. However, there were few studies on the effect of TBM on rapeseed germination.

The germination period is the key stage of growth and development of crops, and it is highly sensitive to external stress [18]. Studies have shown that sulfonylurea herbicide stress during germination could be used to screen plants for tolerant germplasm [19], reducing the impact of TBM on crop production. Germination is a complex process involving specific gene transcription, post-translational modifications, and metabolic interactions [20] that are difficult to analyze by conventional physiological and biochemical methods. This study utilized RNA-seq to detect genes related to TBM stress during the germination stage of *B. napus*, characterize the physiological indices, and verify gene expression by qRT-PCR. The physiological and molecular data were combined to elucidate the response mechanism of rapeseed to TBM stress. This not only improves the accuracy of the results but also provides key information for screening and cultivating TBM-tolerant rapeseed germplasm and exploring the molecular mechanisms of TBM tolerance during germination.

## Results

### Comparison of germinated seed root length between S (sensitive) and R (resistant) *Brassica napus* lines

**Fig. 1:**
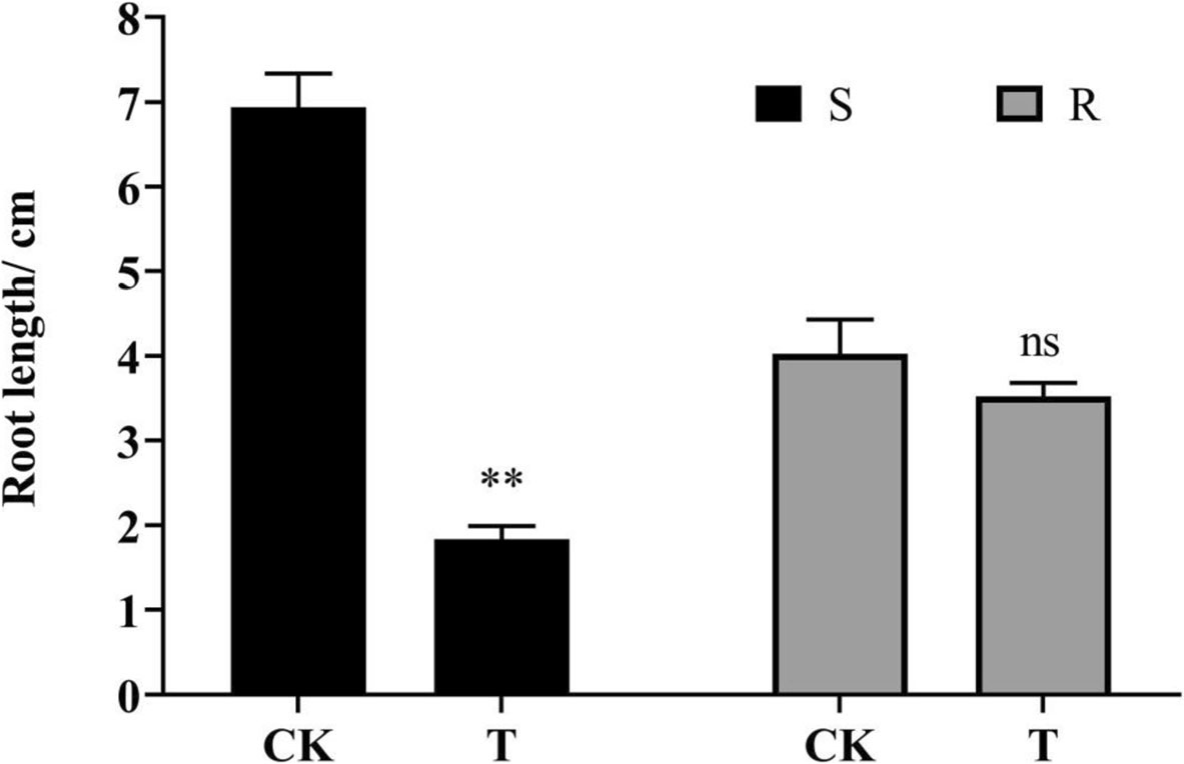
Comparison of root length between different rape lines after 7 d germination. All results are expressed as the mean ± standard deviation (S.D.) of triplicate values. The symbols ‘ns’ and ‘**’ respectively represent ‘not significantly different (*P* > 0.05)’ and ‘an extremely significant difference (0.001 < *P* < 0.01)’, according to Student’s *t*-test

As shown in Fig. 1, the root length of the S line was significantly inhibited after exposure to TBM, while the root length of the TBM-treated R line was no different from control. This indicated that the tolerance of the S and R rapeseed lines to TBM was significantly different from each other.

### Sequencing quality and expression analysis

**Fig. 2:**
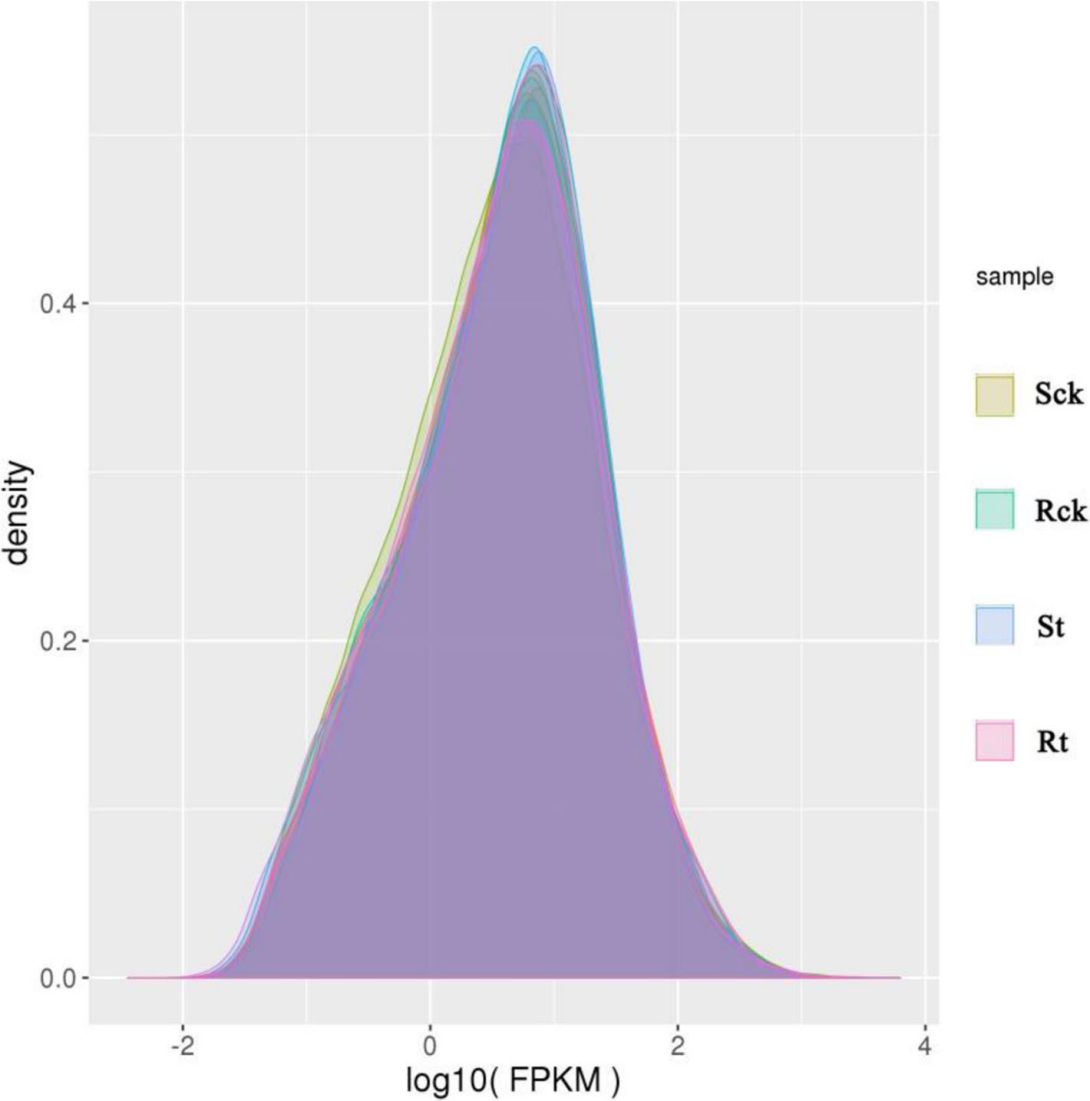
FPKM density distribution of genes in the four simples

45,631,028, 43,758,578, 44,548,434, and 46,766,702 original reads were generated from the four RNA libraries of Sck (S line control), Rck (R line control), St (S line treatment), and Rt (R line treatment), respectively. After removing the low-quality reads, 40,034,436, 38,350,620, 39,237,176, and 42,615,278 high-quality reads were sequentially generated. The percentage alignment of the high-quality reads with the *Brassica* reference genome sequence was 82.28–84.6%. The percentages of single comparisons and multiple comparisons were 95.33–95.55% and 4.45–4.67%, respectively. Q20 and Q30, the percentages of bases with a correct base recognition rate greater than 99.0–99.9% were 94.43–95.5% and 88.17–88.58%, respectively, and the percentage of fuzzy bases (N) was no higher than 0.0046% (Table S1). FPKM density distribution showed that moderately expressed genes accounted for the vast majority, while weakly expressed and highly expressed genes were in the minority (Fig. 2).

### Differentially expressed gene (DEG) analysis

**Fig. 3:**
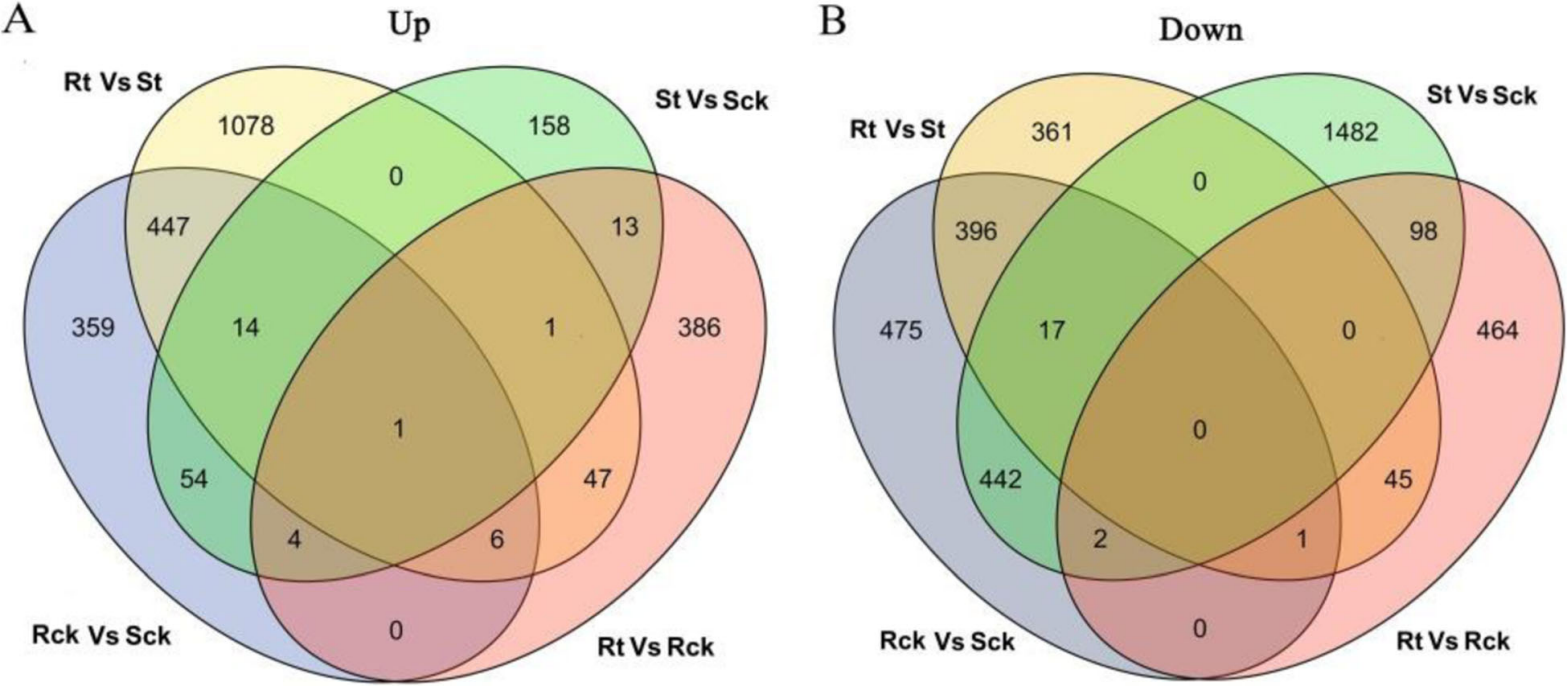
Venn diagram of the number of DEGs detected in four simples. **a.** Venn diagram indicated the number of up-regulated DEGs. **b.** Venn diagram indicated the number of down-regulated DEGs

**Fig. 4:**
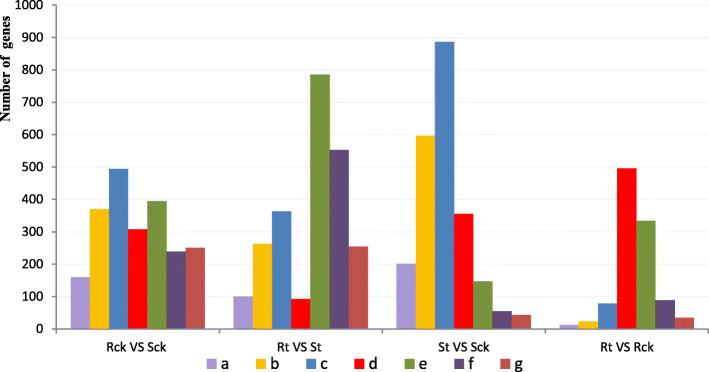
log_2_fold change in the DEGs detected in Rck VS Sck, Rt VS St, St VS Sck and Rt VS Rck. **a.** Number of genes with a log_2_fold change ≤ −5. **b.** Number of genes with −5 < log_2_fold change ≤ −3; **c.** Number of genes with −3 < log_2_fold change ≤ −2. **d.** Number of genes with −2 < log_2_fold change ≤ −1. **e.** Number of genes with 1 ≤ log_2_fold change < 3; **f.** Number of genes with 3 ≤ log_2_fold change < 5; **g.** Number of genes with log_2_fold change ≥5

As shown in Fig. 3 and Fig. 4, a total of 2218 DEGs was obtained from Rck vs. Sck. The number of down-regulated DEGs (1333, 60.1%) was more than that of up-regulated DEGs (885, 39.9%). 2414 DEGs were identified in Rt vs. St, including 1594 (66.0%) up-regulated genes and 820 (34.0%) down-regulated genes, and the log_2_ fold-change of most DEGs was approximately + 1 to + 5. In St vs. Sck and Rt vs. Rck, 2286 and 1068 DEGs were detected, respectively. Of the 2286 DEGs in the S line, 245 (10.7%) were up-regulated and 2041 (89.3%) were down-regulated, and the log_2_ fold-change of most DEGs ranged from − 5 to − 1. The 1068 DEGs of the R line included 458 (42.9%) up-regulated genes and 610 (57.1%) down-regulated genes. The log_2_ fold-change was between − 2 and 3.

### Enrichment analysis of DEGs in Rt vs. St, St vs. Sck and Rt vs. Rck

**Fig. 5:**
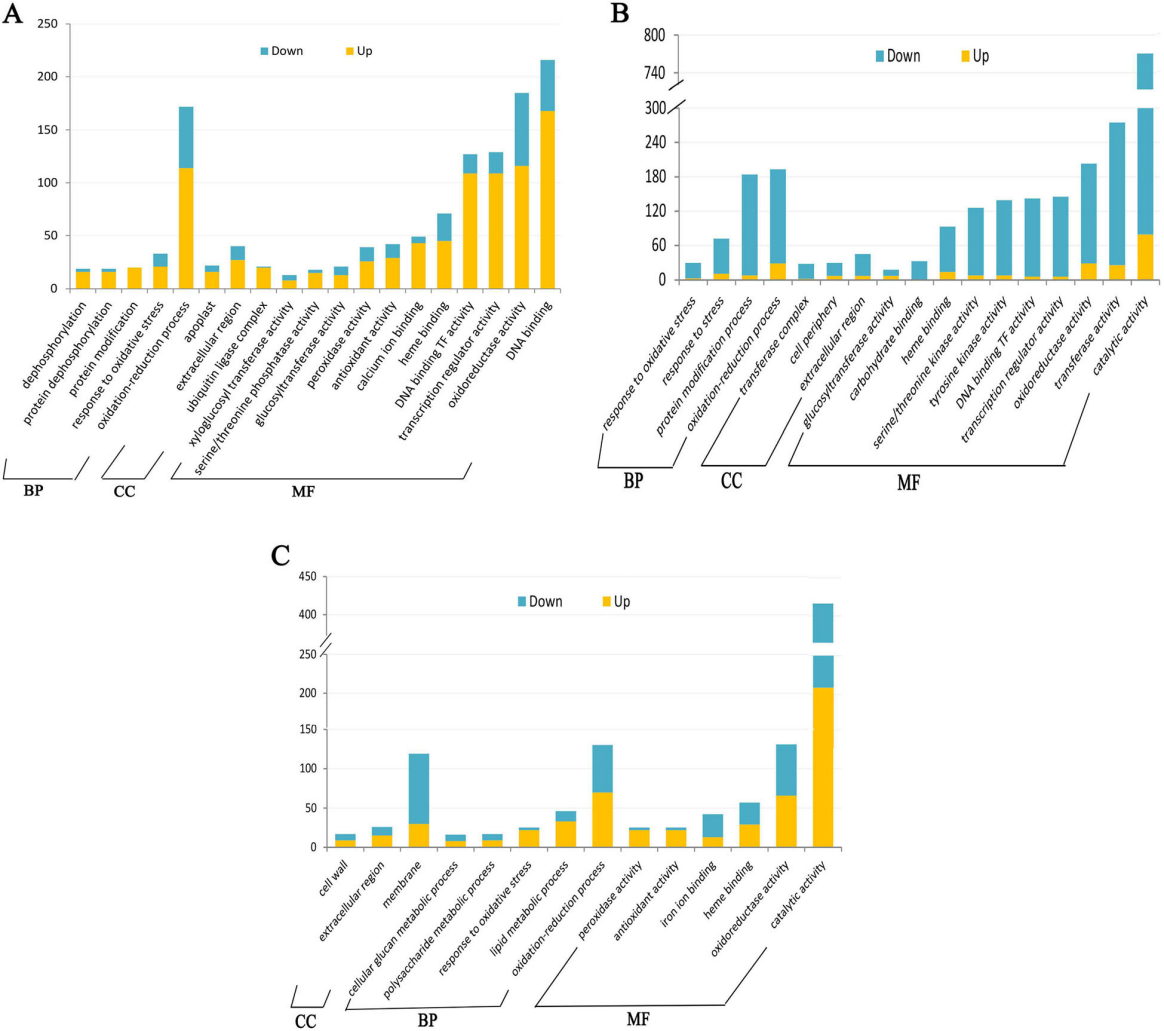
GO classification of DEGs. **a**. GO classification of DEGs in Rt VS St. **b.** GO classification of DEGs in St VS Sck. **c.** GO classification of DEGs in Rt VS Rck. BP: biological process; MF: molecular function; CC: cellular component. The x-axis represents the most abundant categories of each group, and the y-axis represents the number of the total genes in each category

The DEGs in Rt vs. St, St vs. Sck and Rt vs. Rck were annotated into 19, 17 and 14 significant GO terms, respectively (Fig. 5). Under biological processes, oxidation-reduction reactions were overrepresented in Rt vs. St, St vs. Sck and Rt vs. Rck. DEGs in the S and R lines were annotated for responses to oxidative stress. Under cellular components, ubiquitin ligase complex, extracellular region, and apoplast were the most abundant terms in Rt vs. St; and DEGs in the S and R lines were mainly annotated to the extracellular region and membranes, respectively. As for molecular functions, the DEGs in the three groups were mainly related to oxidoreductase activity. In addition, DEGs in Rt vs. St were also involved in transcriptional regulation and DNA binding, and DEGs in the S and R lines participated in catalytic activity.

KEGG enrichment was done to identify in which metabolic pathways the DEGs were involved. As shown in Table 1, the DEGs in Rt vs. St were significantly enriched in phenylpropanoid biosynthesis, cysteine and methionine metabolism, plant-pathogen interaction, MAPK signaling, alpha-linolenic acid metabolism, and linoleic acid metabolism. The DEGs in the S and R lines were significantly enriched in 18 and 9 metabolic pathways, respectively and five pathways were shared by both S and R lines, including phenylpropanoid biosynthesis, alpha-linolenic acid metabolism, tyrosine metabolism, plant hormone signal transduction, cysteine, and methionine metabolism. There were 13 unique pathways in the S line, including plant-pathogen interactions, glucosinolate biosynthesis, and MAPK signaling, while four unique pathways including valine, leucine and isoleucine degradation were found in the R line.

**Table 1:** KEGG pathways were significantly enriched in each group

Pathway ID	Pathways	Up	Down	***P***-value	FDR
Rt VS St					
bna04626	Plant-pathogen interaction	55	8	8.78E-15	9.57E-13
bna00940	Phenylpropanoid biosynthesis	30	17	9.99E-09	5.44E-07
bna04016	MAPK signaling pathway - plant	29	7	2.93E-07	1.06E-05
bna00592	alpha-Linolenic acid metabolism	12	2	0.0001474	0.0040162
bna00591	Linoleic acid metabolism	6	0	0.0010908	0.0237799
bna00270	Cysteine and methionine metabolism	18	5	0.0016719	0.0303735
St VS Sck					
bna04626	Plant-pathogen interaction	2	62	5.23E-20	4.81E-18
bna00966	Glucosinolate biosynthesis	0	17	4.69E-15	2.16E-13
bna00940	Phenylpropanoid biosynthesis	5	46	4.25E-14	1.30E-12
bna04016	MAPK signaling pathway - plant	2	32	1.39E-08	3.19E-07
bna00592	alpha-Linolenic acid metabolism	0	14	1.40E-05	0.0002576
bna00270	Cysteine and methionine metabolism	3	21	2.91E-05	0.0004459
bna04075	Plant hormone signal transduction	4	45	4.10E-05	0.0005385
bna00400	Phenylalanine, tyrosine and tryptophan biosynthesis	1	13	8.44E-05	0.0009708
bna00480	Glutathione metabolism	0	19	0.0003907	0.0039938
bna00380	Tryptophan metabolism	2	12	0.0006111	0.0056225
bna00750	Vitamin B6 metabolism	1	4	0.0016192	0.0135421
bna00591	Linoleic acid metabolism	0	5	0.0024901	0.0190908
bna00920	Sulfur metabolism	3	6	0.0029768	0.0210665
bna00906	Carotenoid biosynthesis	0	8	0.0050363	0.0310883
bna00950	Isoquinoline alkaloid biosynthesis	1	6	0.0050687	0.0310883
bna00360	Phenylalanine metabolism	1	9	0.0076583	0.0415345
bna00430	Taurine and hypotaurine metabolism	0	6	0.0076748	0.0415345
bna00350	Tyrosine metabolism	1	8	0.0084495	0.0431863
Rt VS Rck					
bna00940	Phenylpropanoid biosynthesis	29	6	5.97E-12	5.25E-10
bna00592	alpha-Linolenic acid metabolism	3	9	1.33E-06	5.83E-05
bna00350	Tyrosine metabolism	5	4	0.0002227	0.0061383
bna04075	Plant hormone signal transduction	6	25	0.000279	0.0061383
bna00270	Cysteine and methionine metabolism	6	9	0.0004649	0.00767
bna00520	Amino sugar and nucleotide sugar metabolism	13	4	0.000523	0.00767
bna00280	Valine, leucine and isoleucine degradation	0	9	0.0007443	0.0093566
bna00062	Fatty acid elongation	7	0	0.0009906	0.0108961
bna00250	Alanine, aspartate and glutamate metabolism	5	4	0.0036709	0.0358928

### Functional classification of DEGs

**Fig. 6:**
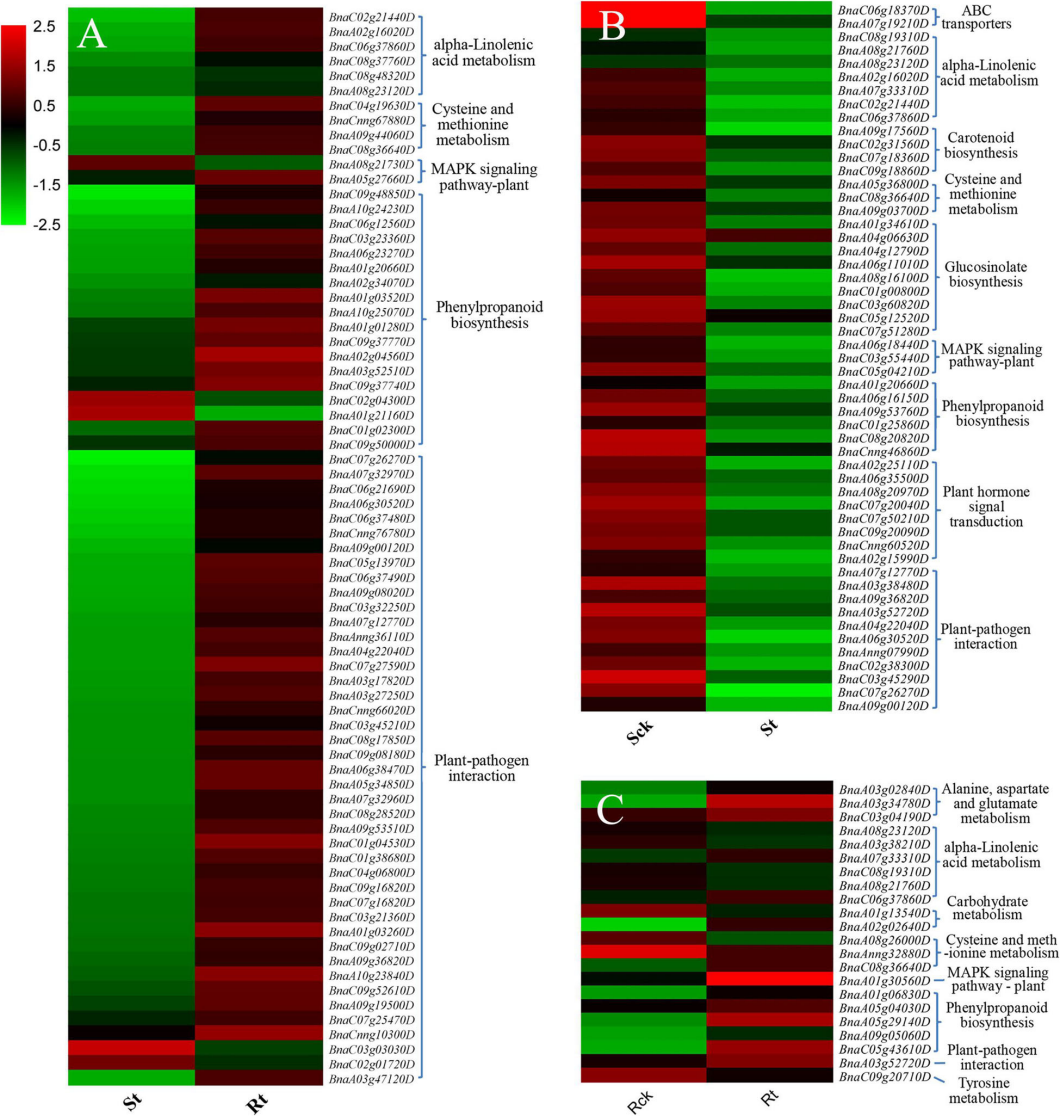
Expression analysis of DEGs related to tribenuron-methyl in the four samples. **a.** Heatmap of DEGs in Rt VS St. **b.** Heatmap of DEGs in St VS Sck. **c.** Heatmap of DEGs in Rt VS Rck

**Fig. 7:**
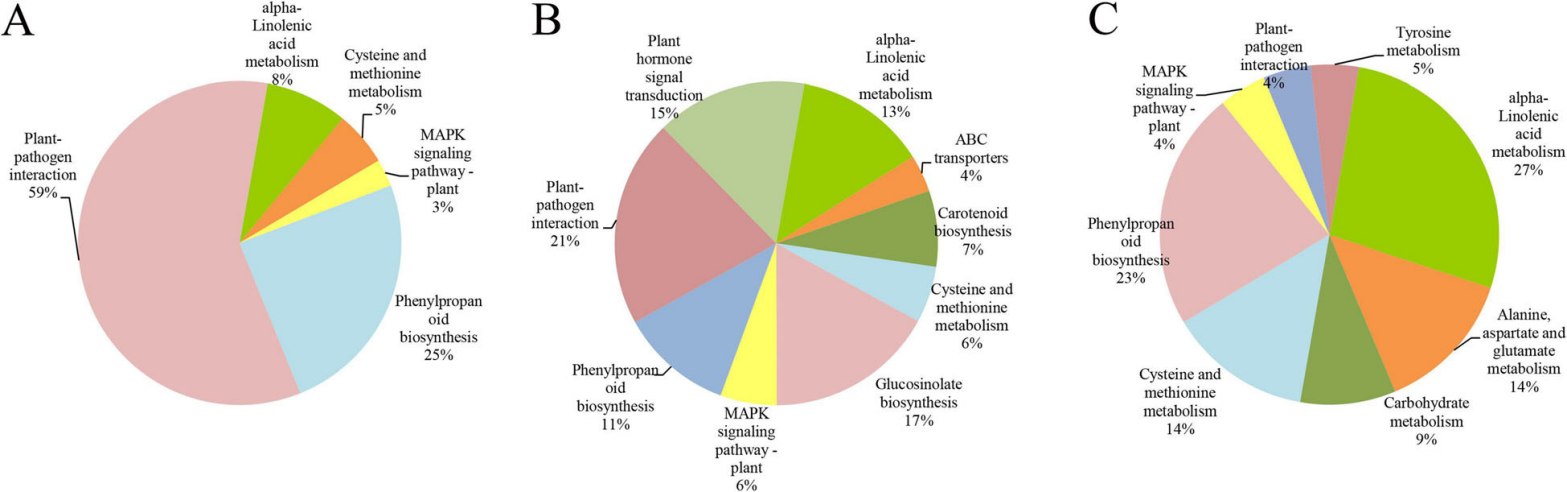
Classification of metabolic levels of DEGs related to tribenuron-methyl. **a.** Classification of metabolism levels of DEGs in Rt VS St. **b.** Classification of metabolism levels of DEGs in St VS Sck. **c** Classification of metabolism levels of DEGs in Rt VS Rck. The digital numbers represent the ratios of genes in different category to all DEGs. Different colors denote different gene clusters

Combining GO and KEGG enrichment analysis (Table S2), 73 DEGs were identified in Rt vs. St, and most of these genes were expressed at higher levels in Rt after TBM treatment (Fig. 6a). These genes were involved in five metabolic pathways with 59% of the genes being related to plant-pathogen interactions and 25% related to phenylpropanoid biosynthesis (Fig. 7a). Screening revealed 53 DEGs from the S line and 22 DEGs from the R line. As shown in Fig. 6b-c, the majority of DEGs were down-regulated in the S line and up-regulated in the R line after treatment. The DEGs in the S line were associated with nine metabolic pathways, including plant-pathogen interaction (21%), glucosinolate biosynthesis (17%) and plant hormone signal transduction (15%) (Fig. 7b). The DEGs in the R line were associated with eight metabolic pathways, and the top three most enriched metabolic levels were alpha-linolenic acid metabolism (27%), phenylpropanoid biosynthesis (23%) and cysteine and methionine metabolism (14%) (Fig. 7c).

**Fig. 8:**
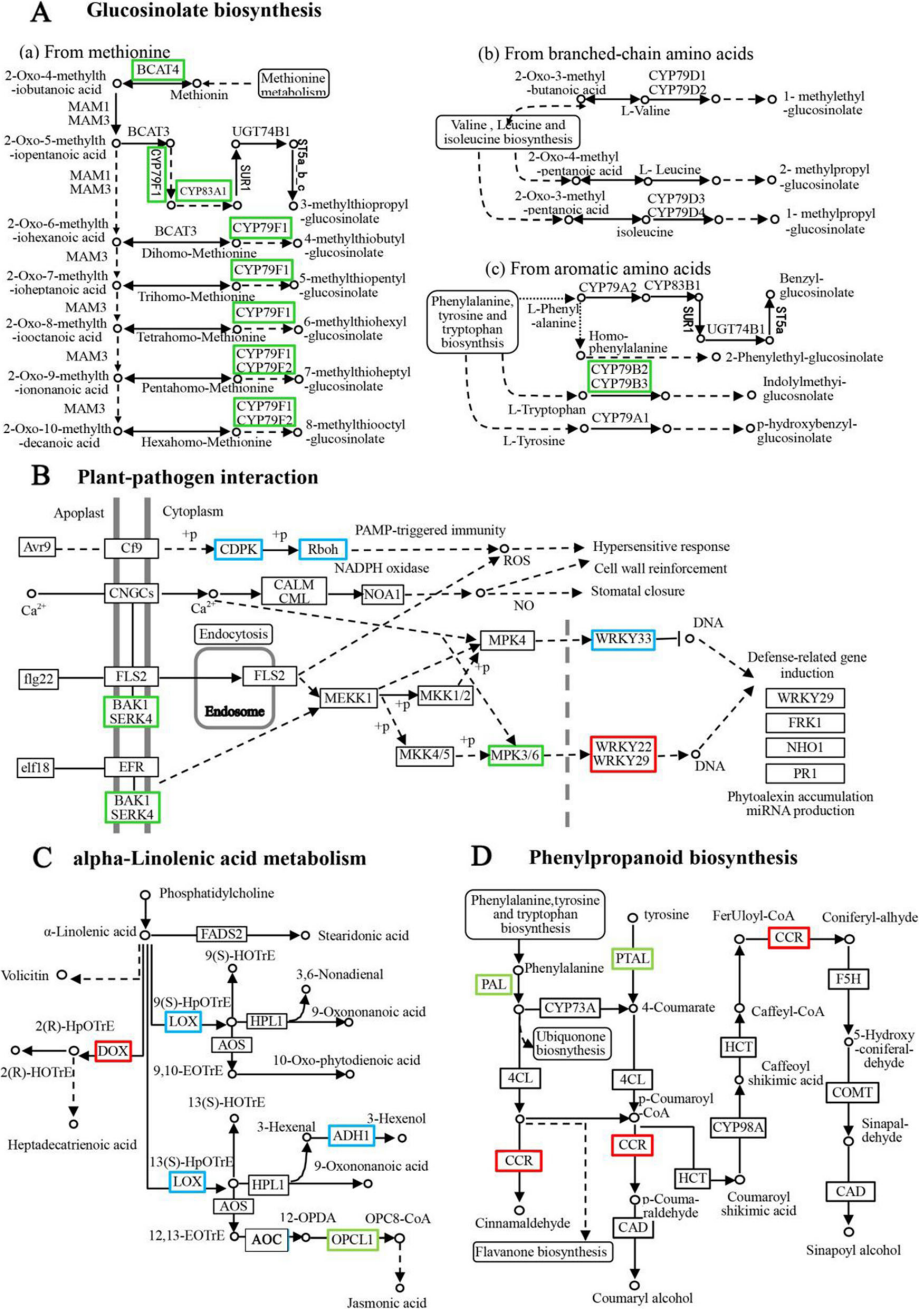
Metabolic pathway in which some DEGs related to tribenuron-methyl were involved. Boxes generally represent enzymes, small circles represent metabolites, and rounded boxes represent another metabolic pathway diagram. Green represents down-regulated gene, red represents up-regulated gene, and blue represents both up- and down-regulated gene. The expression of gene in this figure refers to comprehensive performance among in Rt VS St, St VS Sck and Rt VS Rck

In Rt vs. St, there were 43 genes encoding calmodulin-like (CML) proteins, respiratory burst oxidase homolog (RBOH), WRKY DNA-binding protein, calcium-dependent protein kinase (CPK), calcium-binding EF-hand family proteins and the disease-resistance protein family, which influence plant-pathogen interactions. As shown in the metabolic pathway (Fig. 8b), CDPK affects the expression of RBOH by sensing the Ca^2+^ level, thereby stimulating the generation of ROS. WRKY22 and WRKY33 induce the expression of defense-related genes, eventually reorganizing the cell wall or inducing hypersensitivity. Genes encoding lipoxygenase 3 (LOX3), allene oxide cyclase 3 (AOC3), PLAT/LH2 domain-containing lipoxygenase family protein and alcohol dehydrogenase (ADH1) were enriched in α-linolenic acid metabolism (Fig. 8c), and > 4-fold changes of these genes were induced in Rt relative to St. Peroxidase-related genes were found in phenylpropanoid biosynthesis. They produced H_2_O_2_ during the defense reaction, which in turn stimulated an antioxidant stress response (Fig. 8d).

The genes encoding RBOH, WRKY, LOX3, ADH1, ACO1, peroxidase, and calcium-dependent protein were down-regulated in the S line. In the R line, however, RBOH, WRKY, and calcium-dependent protein were not detected, while the genes encoding ADH1, ACO1 and peroxidase were up-regulated (Fig. 6b-c). The genes encoding CYP79F1, CYP83A1, CYP79B2, CYP79B3 and BCAT4, which are secondary metabolites that contribute to plant defense, were found in the glucosinolate biosynthetic pathway (Fig. 8a); the genes encoding MPK3 and CDPK were detected in the signal transduction and plant-pathogen interaction pathways. In these pathways, MPK family genes stimulate the expression of WRKY family members and ultimately affect the expression of related defense genes in the S line (Fig. 8b). In general, there were many DEGs between the S and R lines after TBM exposure. Combining GO and KEGG enrichment analysis, the DEGs were all down-regulated in the S line, but about 70% of the R line DEGs were up-regulated, suggesting that TBM can have an adverse reaction on rapeseed by inhibiting the biosynthesis of secondary metabolites, disrupting lipid metabolism or cell membrane structure and influencing stress signal transduction. These results also explain why the root system of S line plants was more severely inhibited compared to R line.

### Verification of gene expression data by qRT-PCR analysis

**Fig. 9:**
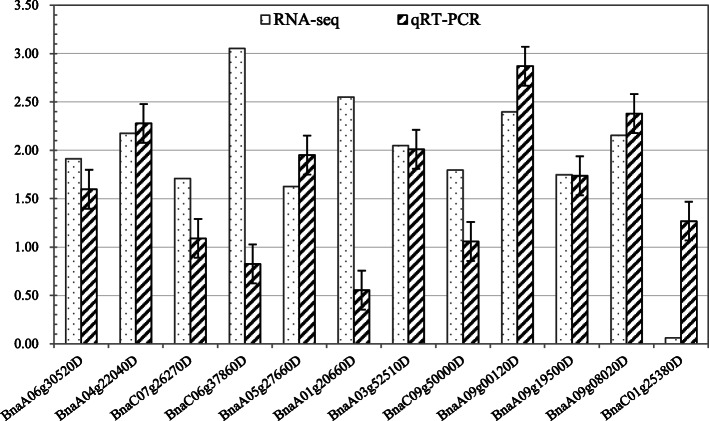
qRT-PCR verification analysis. The qPCR values are presented as the averages of triplicates

To verify the RNA-seq results, 11 genes were randomly selected from the 73 genes identified above in Rt vs. St and subjected to qRT-PCR analysis. We also performed qRT-PCR to confirm expression of ALS isozyme genes (BnaC01g25380D) to distinguish expression levels between R and S lines. As shown in Fig. 9, the results of qRT-PCR analysis were consistent with the RNA sequence data, highlighting the reliability of the RNA-sequencing procedure.

### Measurement of physiological parameters

**Fig. 10:**
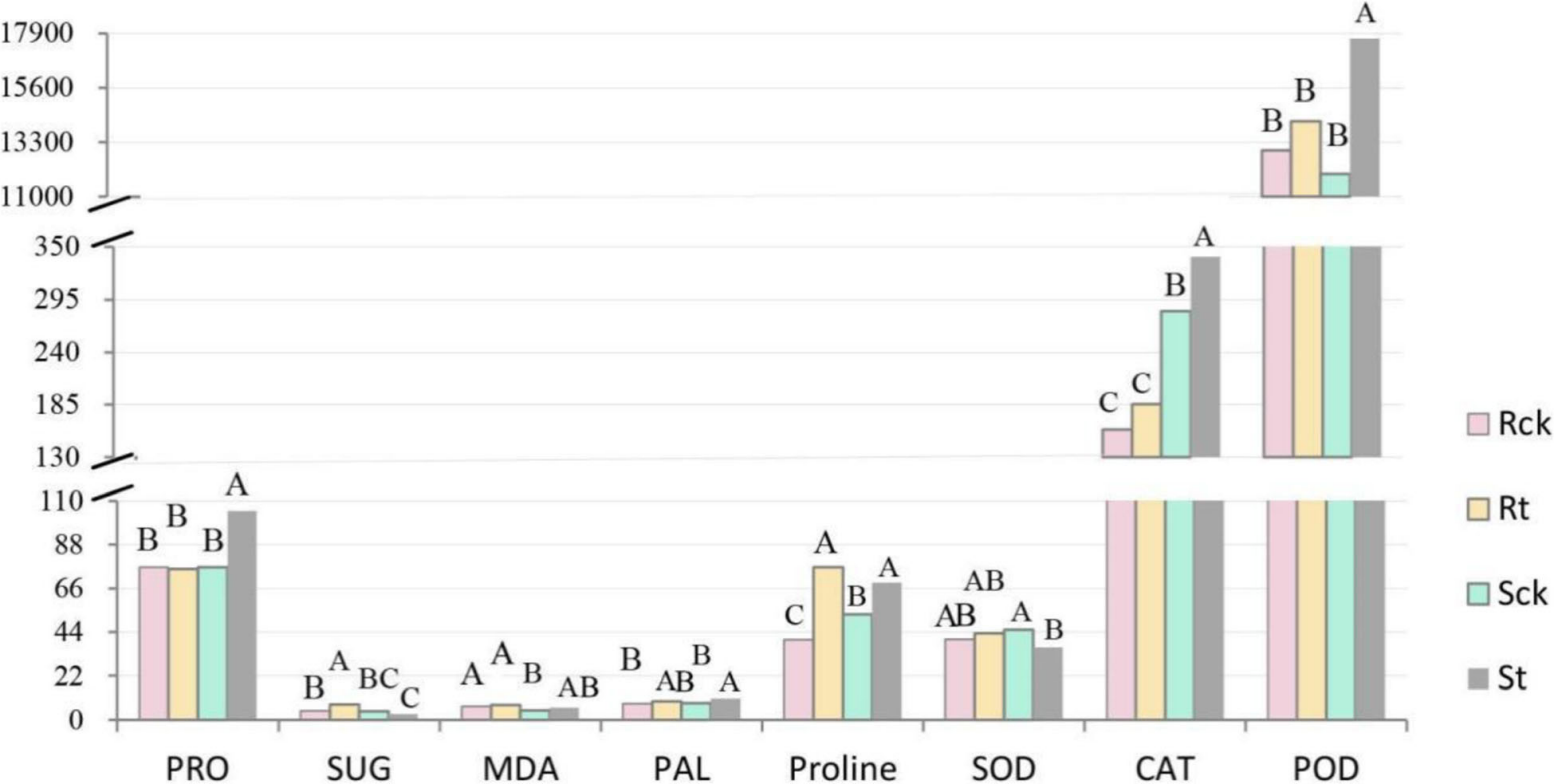
Changes of 8 physiological indicators in four simples. Each bar shows averages of triplicates. **a**, **b** and **c** represent significant difference (*P*<0.05). SUG: soluble sugar; MDA: malondialdehyde; PAL: phenylalanine ammonia-lyase; SOD: superoxide dismutase; CAT: catalase; POD: peroxidase

Most of the genes found by screening above were redox-related, so we also analyzed a few relevant physiological indicators (Fig. 10). Carbohydrate and protein are the main raw materials of metabolism during seed germination and in this study, SUG and PRO content in the Sck and Rck were almost the same. After treatment, the content of SUG in the Rt increased significantly by 72.6% compared to control, while that in the St decreased by 33.8%. The PRO content in the St increased significantly by 37% comparing with control, and it was significantly higher than that of other treatments (*p* < 0.05); but, the PRO content of the R line did not change significantly. Environmental stressors can promote the production of harmful metabolites such as reactive oxygen species (ROS) in plants. Oxygen free radicals act on unsaturated fatty acids to produce lipid peroxides that break down to form toxic compounds like malondialdehyde (MDA) [7]. Compared to the control, the MDA content of the St and Rt increased by 28.9 and 13.1%, respectively. Proline protects membranes and enzymes from ROS damage [9], and superoxide dismutase (SOD), catalase (CAT), and peroxidase (POD) are important components of the antioxidant enzyme system that keeps oxidation reactions in check [21]. Our data showed that the proline content of the St and Rt lines significantly increased by 30.3 and 91.2%, respectively (*p* < 0.05). The SOD level in St significantly decreased by 19.58%, and the activities of CAT and POD significantly increased by 20 and 47.96%, respectively. The activity of SOD, CAT and POD in Rt was not significantly different from the control group. However, there were significant differences in CAT and POD between St and Rt. Phenylalanine ammonia lyase (PAL) is a key enzyme in the phenylpropanoid pathway [22], which is involved in the stress response. In this study, the PAL activity in St significantly increased by 29.3% (*p* < 0.05), while that of Rt only increased by 13.8%.

## Discussion

The response to herbicide stress involves various antioxidant defense mechanisms [15], including enzymatic and non-enzymatic antioxidant detoxification pathways. The most common non-enzymatic antioxidant pathways include the defense response activated by plant hormones and signal transduction, as well as osmotic regulation [12]. Herbicide resistance mechanisms include mutations in target-site resistance (TSR) genes and non-target-site resistance (NTSR) changes. ALS is the first enzyme in the biosynthetic pathway of branched-chain amino acids [23]. Although we did not detect a significant change in ALS activity or that of other genes in form branched-chains amino acids of the glucosinolate biosynthetic pathway, we assume that ALS expression did occur. Studies have shown that herbicide damage to plants begins 3–8 h after herbicide application [24], and the expression level of ALS might be inhibited during that time. With increasing exposure time, the gene expression in the different lines returned to similar levels, and no difference was detected. This was confirmed by qRT-PCR for ALS isozymes. TBM mainly affects biosynthesis of branched-chain amino acids, and the two genes encoding branched-chain aminotransferase (BCA) in the S and R lines were strongly down-regulated by TBM treatment, implicating a probable negative regulation function in the adaptation to TBM.

Apart from the above-mentioned TSR genes, increasing herbicide metabolic rate is an important mechanism for enhancing herbicide resistance. The main biotransferases involved in herbicide metabolism include cytochrome P450 monooxygenase (P450), ATP binding cassette (ABC) transporter, and glutathione S-transferase (GST) [13, 25]. In this study, nine genes encoding CYP450 (CYP83A1, CYP79F1, CYP79B2, CYP79B3), which are key enzymes in the glucosinolate biosynthetic pathway, were identified as DEGs down-regulated only in the S line. Among them, CYP83A1 [26] and CYP79F1 [27] were involved in the metabolism of toxic substances and CYP79B2 and CYP79B3 could convert tryptophan to IAA (indole-3-acetic acid) [28]. Carotenoid biosynthesis in plants could be inhibited by herbicides at different steps [29]. We found four differentially expressed CYP707A3 genes that were strongly down-regulated in the S line by TBM treatment. CYP707A3 encodes ABA 8′-hydroxylase [30]. Previous studies have shown that increased expression of HvABA8’OH1 was associated with a higher germination rate [31]. This was consistent with our research, in which the expression of ABA 8′-hydroxylase was inhibited in the S line, and germination ability and root length were reduced. GST catalyzes the reaction between herbicide and glutathione, or acts as a peroxidase to reduce oxidative damage [15]. The active site, Cys-19 in GSTZ1, has an especially strong catalytic effect [32]. In previous studies, the GST gene was found to have been mostly up-regulated after herbicide treatment [13, 33, 34]. Our study identified a gene encoding GSTZ1, which was down-regulated in the R line. The reason for this difference might be that the phi-, tau- or lambda-like GSTs are much more responsive to chemical treatments, but zeta-like GST genes are not strongly induced by these treatments [35]. The ABC transporter is an efflux pump located on the cell membrane and an important protector against external stresses [36]. In general, the transcription of the ABC transporter was significantly increased after herbicide treatment [12, 13, 37], however, our results differ from these findings. Over the seven-day exposure period, TBM caused the gene encoding ABCB5 to be down-regulated in the S line but no significant change in the R line. The reason for this could be the different genetic background of the two rapeseed lines, where the expression of genes related to detoxification was suppressed in the S line, while the R line retained the capacity to breakdown TBM. This result demonstrated that the R line had a greater ability to promote expression of genes related to detoxification so that it could prevent damage from TBM.

As part of the defense mechanism, plant hormones play an important role in resistance by activating secondary signals [38, 39]. Previous researches showed that the expression of jasmonate promoted resistance [40]. BRs are critical for the induction of the detoxifying response against herbicides [41]. Overexpression of GmBIN2 in transgenic roots resulted in significantly higher relative root growth than in controls under abiotic stress [39]. In this study, no hormone-related genes such as jasmonate or brassinosteroids (BRs) were detected in the R line, but eight down-regulated genes encoding JAZ6, BIN2 and ERF were found in the S line. Auxin-based herbicides might directly induce the expression of ACC oxidase, and plant resistance could be associated with reduced ethylene synthesis [42, 43]. After TBM treatment, six genes encoding ERF2 were down-regulated in the S line, while few genes were detected in the R line. This gene expression alteration in the S line was consistent with the TBM-sensitivity phenotype. In addition, the expression of ACC oxidase was down-regulated in the S line but up-regulated in the R line, with log2 fold-changes of − 1.73 and 1.58, respectively. We inferred that expression of the ACC oxidase gene was slightly up-regulated in the R line, which might be related to the delayed response of tolerant varieties to TBM, or the existence of some other regulatory mechanism, like negative feedback regulation. A similar result was reported by Gao [44].

In addition to plant hormones, we also found some specific changes in expression of genes related to antioxidant stress. A group of 19 genes encoding WRKY and RBOH and 29 calmodulin-related genes were also found in the plant-pathogen interaction pathway that is part of the signal transduction attribute. Previous studies showed that CDPK [45], WRKY [46], Rboh [47, 48], and MAPKs could regulate plant tolerance by activating and regulating gene expression, transmitting ROS signals, and triggering hydrogen peroxide-induced antioxidant enzyme activity [14]. In the MAPK signaling pathway, two MAPK-related genes and one CAT-related gene were identified in the S line and R line, respectively. In addition, 23 genes related to POD were screened as part of the phenylpropanoid biosynthetic pathway. POD-related genes produce H_2_O_2_, which stimulates the antioxidant stress response. These genes play an important role in enhancing plant resistance. For example, WRKY is from a group of transcription factors that play a vital role in stress tolerance. Their expression was higher in Rt than in St, and similar results were obtained by other researchers who found contrasting expression pattern of genes like WRKY in resistant and susceptible genotypes [49, 50]. These genes do not work independently, as shown in the metabolic pathway (Fig. 8). CDPK alters the expression of RBOH by sensing the Ca^2+^level. RBOH reduces molecular oxygen to superoxide, and the latter is converted into H_2_O_2_ by superoxide dismutase. MAPK family genes stimulate the expression of WRKY family members and ultimately affect the expression of related defense genes. Previous studies have shown that the ROS scavenging system could be triggered by herbicide stress to promote the metabolism of herbicide molecules [21, 44, 51]. In this study, the activity of CAT, POD and PAL increased after TBM exposure, but the increase in the S line was higher than that of the R line. In contrast, SOD activity decreased in the S line but increased in the R line. This was consistent with the transcriptome results and phenotypic characteristics (Fig. 1), as well as previous results [38]. Taken together, these changes demonstrated that TBM stress inhibited the expression of stress-related genes and led to accumulation of toxic substances in the S line, while the R line was able to up-regulate gene expression to counteract ROS.

Herbicides have been reported to impair carbon metabolism leading to accumulation of carbohydrates [52–54]. In the R line, genes encoding UDP-glucose/UDP-galactose 4-epimerase (UGE4) and UDP-glucose dehydrogenase (UGD) were found, which were annotated to carbohydrate metabolism. UGE4 mediates the conversion of UDP-glucose and UDP-galactose [55], while UGD converted UDP-glucose to UDP-glucuronic acid [56], which is used for cell wall carbohydrate biosynthesis. A previous study showed that carbohydrate accumulated in roots as a result of herbicide treatment [54]. In this study, SUG content in the R line increased by 73%, but in the S line it decreased by 34%, compared to control. Another common effect on the roots of plants treated with herbicide was the induction of fermentation, but an increase in ADH activity may reduce this effect [57]. Our study showed that genes encoding ADH1 were up-regulated in Rt vs. St and the R line but down-regulated in the S line. As expected for ADH1-related genes, those encoding LOX3, ADH1, and OPC-8:0 CoA ligase1 (OPCL1) were up-regulated in Rt vs. St, but down-regulated in the S and R lines. LOX preserved intact cell membranes by delaying breakdown of polyunsaturated fatty acids, thereby reducing the formation of cytotoxic derivatives [58]. OPCL1 took part in the synthesis of JA which regulated defense-related processes in higher plants [59]. The genes mentioned above were also detected in metabolism of the unsaturated fatty acid, alpha-linolenic acid. ROS can react with unsaturated fatty acids in the cell membrane, leading to membrane lipid peroxidation, cell membrane damage, and increased permeability [9]. MDA is an intermediate product of lipid peroxidation [7, 58], and our results showed that the MDA levels in the R and S lines increased by 13 and 29%, respectively. Proline acts as a protective agent for biomembranes and as an osmotic regulator [9]. The proline concentration in the R line increased by 91%, which was much higher than the 30% of the S line. The greater amount of proline produced by the R line may have served to prevent membrane damage from lipid peroxidation and helped to maintain the intracellular osmotic balance, so that only a small amount of MDA was produced; thus, the R line showed greater resistance to TBM-induced damage.

## Conclusion

After TBM stress, most DEGs were down-regulated in the S line but up-regulated in the R line. GO and KEGG analysis revealed 137 genes, including those related to antioxidant stress and herbicide detoxification such as *RBOH, WRKY, CYP450, ABC, MPK3, CDPK, DOX, LOX3, and ADH*. These genes were mainly enriched in the redox pathway and some metabolic pathways such as plant-pathogen interactions, α-linolenic acid metabolism, glucosinolate biosynthesis, and phenylpropanoid biosynthesis. The results were further verified by eight physiological indices. In summary, the resistant and sensitive rapeseed lines both underwent oxidative stress after TBM exposure, but, compared to the S line, the R line was better able to regulate gene expression to reduce the oxidative damage caused by the tribenuron-methyl herbicide.

## Methods

### Plant materials and TBM treatment

The relevant concentration of TBM was ascertained from a search of the literature [60]. *Brassica napa* 27,123 was selected as the sensitive (S) line while 27,085 was chosen as the resistant (R) line from the RIL population containing 172 lines. Twenty seeds of S and R lines were placed on three layers of filter paper moistened with 3 ml of 0.15 mg•kg^− 1^ TBM. Distilled water was used as the control. The seeds were placed into a climate chamber at 25 °C, 85% relative humidity, and 16 h/8 h of light / darkness. Each test was conducted with 20 replicates. At day 7 of treatment, the root lengths were measured and 0.1 g root samples of each biological replicate were collected into 1.5 mL centrifuge tubes, quickly frozen in liquid nitrogen and stored at − 80 °C for later determination of physiological indices and qRT-PCR. The control and treated samples of the S and R lines were labeled Sck and Rck, and St and Rt, respectively. The RIL population came from a cross between 10D130 and Zhongshuang11 (ZS11). 10D130 is a high-generation inbred line selected from the interspecific hybrids of *Brassica juncea* and *Brassica oleracea* by the Chongqing Engineering Research Center, while ZS11 is a conventional high-quality rapeseed variety selected by the Chinese Academy of Agricultural Sciences. The seeds were provided by the Chongqing Engineering Research Center. Tribenuron-methyl (TBM) was the Maifa brand produced by Hetian Chemical Co., Ltd. in Shenyang, China.

### RNA extraction, cDNA library construction, and sequencing

The root samples of S and R lines under control or TBM stress were sent to Personalbio Co., Ltd. (Shanghai, China) for RNA extraction, library construction, and transcriptome sequencing on the Illumina sequencing platform. After removing the 3′-adapter, low-quality sequences (sequence quality values < Q20), the clean data were aligned to the *Brassica napus* reference genome (http://www.genoscope.cns.fr/*Brassica*napus/cgi-bin/gbrowse/colza/) using HISAT2 [61] (http://ccb.jhu.edu/software/hisat2/index.shtml). The read count value was determined by HTSeq [62] (https://htseq.readthedocs.io/en/release_0.11.1/). Fragments per kilobase million (FPKM) values were calculated to estimate gene expression levels. DEGs between the two groups were identified using DESeq [63] based on *p* ≤ 0.05 and |log2 fold-change| ≥ 1. Gene ontology (GO) enrichment analysis of the DEGs was performed using topGO [64], and significantly enriched GO items were selected based on a false discovery rate (FDR) < 0.01. The Kyoto Encyclopedia of Genes and Genomes (KEGG) pathway enrichment analysis was performed using the KOBAS2.0 website (http://kobas.cbi.pku.edu.cn/home) [65], and significant enrichment was selected based on a FDR < 0.01. KEGG database is developed by Kanehisa Laboratories [66–68], and KEGG pathways and other KEGG materials shown in this article were copyrighted by Kanehisa Laboratories.

### qRT-PCR validation

qRT-PCR was performed on a BioRad CFX96 real-time system using a kit from Vazyme Biotechnology Co., Ltd. (Nanjing, China). The reaction conditions were as follows: 95 °C for 30 s and 40 cycles (95 °C for 10 s, 56 °C for 30 s, 72 °C for 60 s). The 2^-ΔΔCt^ method was used to evaluate the relative expression of genes based on the stable expression level of *BnaActin* 7 [10]. The primer pairs were designed by Vector NTI Advance 11.5.1 software and synthesized by Sangon Biotech (Shanghai, China) (Table 2).

**Table 2:** Primers for qRT-PCR of candidate differentially expressed genes

genes	Primer sequence(5′ - 3′)	
	Forward primer	Reverse primer
BraACTIN 7	GGAGCTGAGAGATTCCGTTG	GAACCACCACTGAGGACGAT
BnaA06g30520D	ACCGTCTTCTCTGAGGTATGTA	ATGCCAAGACCTACTAGGAGTA
BnaA04g22040D	GTGCAGACAACAAGTGACATAG	TCACCGCTCTCATATCATTTGA
BnaC07g26270D	TTGTATCTGGGACACGTGTTAA	TTTTAGTTCCTTAGTCGGTGCT
BnaC06g37860D	GAATCGAGATTCTCCATCAACG	GCAACATTCAAAGTAGCTCCAA
BnaA05g27660D	ATGTGCCTTCAAGACTCCGATA	CTCCTCCTTTTCCTCAAGTCAA
BnaA01g20660D	CATCGTACGAGAAACCATTGTC	ATATCTGCGCATGAAACAGTTC
BnaA03g52510D	CTCCAGCGACTAGGAATATTGT	AATTTTTACGGACGTCACCTTG
BnaC09g50000D	TACAACGAGACAAACATCAACG	AAAAATTAGCGGAGTTGACGTC
BnaA09g00120D	GATGTTCATCGTCACTTACACG	TATCCGACAAAGACAGCAGATC
BnaA09g19500D	CGATTCTCCCCGACCTCAAC	CCGTTAGAATCAGCCTCCGT
BnaA09g08020D	CGATTGATAGCAACACTGATGG	CTCTGAGTCATGTTCTTCCAGT
BnaC01g25380D	CGACAAGAACAAGACTTTCGTC	GATAAGCAAAGACGGTTTCGAC

Note: BnaC01g25380D encodes ALS isozyme

### Measurement of physiological parameters in roots

The physiological parameters, including soluble protein (PRO), soluble sugar (SUG), malondialdehyde (MDA), proline content, and phenylalanine ammonia-lyase (PAL), superoxide dismutase (SOD), catalase (CAT), and peroxidase (POD) activities were measured. All measurements were performed in triplicate and means were calculated for further analysis. The proline content was estimated using the method described by predecessors [69]. The contents of PRO, SUG, MDA, PAL, SOD, CAT, and POD were measured using kits from Sino Best Biological Technology Co., Ltd. (Shanghai, China).

## Supplementary Information

**Additional file 1 Table S1.** Quality and annotation of RNA-seq assembly.

**Additional file 2 Table S2.** Genes identified by combined GO and KEGG enrichment analysis.

## Data Availability

The raw sequence data are available in the NCBI Sequence Read Archive (SRA) repository. The accession number is PRJNA717988, and SRA RunSelector as follows: https://www.ncbi.nlm.nih.gov/Traces/study/?acc=PRJNA717988. All data supporting the conclusions of this article are included in the article and its additional files.

## Abbreviations

SNP: single nucleotide polymorphism
DEGs: differentially expressed genes
FPKM: fragments per kilobase million
GO: Gene ontology
KEGG: Kyoto Encyclopedia of Genes and Genomes
PRO: soluble protein
SUG: soluble sugar
MDA: malondialdehyde
PAL: phenylalanine ammonia-lyase
SOD: superoxide dismutase
CAT: catalase
POD: peroxidase
DOX: dioxygenases
LOX3: lipoxygenase 3
ADH1: alcohol dehydrogenase 1
RBOH: respiratory burst oxidase homologue
WRKY: WRKY DNA-binding protein
ACO1: ACC oxidase 1
CYP450: cytochrome P450
ABC: ATP binding cassette subfamily
BCAT4: branched-chain aminotransferase 4
MPK3: mitogen-activated protein kinase 3
CDPK: calcium-dependent protein kinase
ERF2: ethylene-responsive element-binding factor 2
OPCL1: OPC-8:0 CoA ligase1
