# Supplementary material for: Transcriptome analysis reveals gene responses to herbicide, tribenuron methyl, in Brassica napus L. during seed germination
Source: BMC Genomics. 2021 Apr 23;22:299. doi: 10.1186/s12864-021-07614-1 (PMC8067372; doi:10.1186/s12864-021-07614-1)
Supplement: Supplementary file 1 — Additional file 1 Table S1. Quality and annotation of RNA-seq assembly. [file 12864_2021_7614_MOESM1_ESM.docx]

Table S1 Quality and annotation of RNA-seq assembly

| Sample | Sck | Rck | St | Rt |
| --- | --- | --- | --- | --- |
| Reads No. | 45631028 | 43758578 | 44548434 | 46766702 |
| Clean Reads No. | 40034436（87.73%） | 38350620（87.64%） | 39237176（88.07%） | 42615278（91.12%） |
| Q30 (%) | 88.24 | 88.32 | 88.58 | 88.17 |
| Q20 (%) | 94.43 | 94.47 | 94.63 | 95.5 |
| fuzzy bases (%) | 0.002082 | 0.002125 | 0.002265 | 0.00463 |
| Total_Mapped | 33869615 (84.60%) | 31555651 (82.28%) | 33047043 (84.22%) | 35800137 (84.01%) |
| Multiple_Mapped | 1507193 (4.45%) | 1404792 (4.45%) | 1541754 (4.67%) | 1655821 (4.63%) |
| Uniquely_Mapped | 32362422 (95.55%) | 30150859 (95.55%) | 31505289 (95.33%) | 34144316 (95.37%) |
