# Supplementary material for: Transcriptome analysis reveals gene responses to herbicide, tribenuron methyl, in Brassica napus L. during seed germination
Source: BMC Genomics. 2021 Apr 23;22:299. doi: 10.1186/s12864-021-07614-1 (PMC8067372; doi:10.1186/s12864-021-07614-1)
Supplement: Supplementary file 2 — Additional file 2 Table S2. Genes identified by combined GO and KEGG enrichment analysis. [file 12864_2021_7614_MOESM2_ESM.docx]

Table S2 Genes identified by combining GO and KEGG enrichment analysis

| group | Gene ID | AGI number | St:fpkm | Rt:fpkm | log2FC(Rt/St) | Regulate | Gene annotation | Pathways |
| --- | --- | --- | --- | --- | --- | --- | --- | --- |
| Rt Vs St | BnaC02g21440D | AT1G72520 | 0.8525875 | 59.001118 | 6.180 | up | PLAT/LH2 domain-containing lipoxygenase family protein | alpha-Linolenic acid metabolism |
|  | BnaA02g16020D | AT1G72520 | 0.3661559 | 31.550598 | 6.497 | up | PLAT/LH2 domain-containing lipoxygenase family protein | alpha-Linolenic acid metabolism |
|  | BnaC06g37860D | AT1G77120 | 55.371069 | 438.94516 | 3.054 | up | alcohol dehydrogenase 1 (ADH1) | alpha-Linolenic acid metabolism |
|  | BnaC08g37760D | AT1G17420 | 0.1126633 | 1.8000341 | 4.066 | up | lipoxygenase 3 (LOX3) | alpha-Linolenic acid metabolism |
|  | BnaC08g48320D | AT1G17420 | 0 | 1.1500218 | Inf | up | lipoxygenase 3 (LOX3) | alpha-Linolenic acid metabolism |
|  | BnaA08g23120D | AT1G17420 | 0 | 1.2750242 | Inf | up | lipoxygenase 3 (LOX3) | alpha-Linolenic acid metabolism |
|  | BnaC04g19630D | AT1G62380 | 3.7940522 | 49.511842 | 3.774 | up | ACC oxidase 2 (ACO2) | Cysteine and methionine metabolism |
|  | BnaCnng67880D | AT2G19590 | 12.400405 | 129.57671 | 3.453 | up | ACC oxidase 1 (ACO1) | Cysteine and methionine metabolism |
|  | BnaA09g44060D | AT2G19590 | 49.992095 | 160.92909 | 1.754 | up | ACC oxidase 1 (ACO1) | Cysteine and methionine metabolism |
|  | BnaC08g36640D | AT2G19590 | 34.994467 | 142.51395 | 2.094 | up | ACC oxidase 1 (ACO1) | Cysteine and methionine metabolism |
|  | BnaA08g21730D | AT1G20620 | 51.141846 | 12.083393 | -2.014 | up | catalase 3 (CAT3) | MAPK signaling pathway - plant |
|  | BnaA05g27660D | AT3G11410 | 48.997117 | 144.24893 | 1.625 | up | protein phosphatase 2CA (PP2CA) | MAPK signaling pathway - plant |
|  | BnaC09g48850D | AT5G06730 | 5.0525901 | 71.756234 | 3.896 | up | Peroxidase superfamily protein | Phenylpropanoid biosynthesis |
|  | BnaA10g24230D | AT5G06730 | 10.24954 | 100.84314 | 3.366 | up | Peroxidase superfamily protein | Phenylpropanoid biosynthesis |
|  | BnaC06g12560D | AT5G39580 | 2.9243025 | 14.276133 | 2.355 | up | Peroxidase superfamily protein | Phenylpropanoid biosynthesis |
|  | BnaC03g23360D | AT2G41480 | 0.2548777 | 5.0525548 | 4.377 | up | Peroxidase superfamily protein | Phenylpropanoid biosynthesis |
|  | BnaA06g23270D | AT5G64120 | 17.878885 | 90.18408 | 2.402 | up | Peroxidase superfamily protein | Phenylpropanoid biosynthesis |
|  | BnaA01g20660D | AT3G49120 | 4.220047 | 23.591876 | 2.551 | up | peroxidase CB (PRXCB) | Phenylpropanoid biosynthesis |
|  | BnaA02g34070D | AT5G64120 | 0.2370052 | 5.1189994 | 4.501 | up | Peroxidase superfamily protein | Phenylpropanoid biosynthesis |
|  | BnaA01g03520D | AT4G33420 | 17.652938 | 73.529518 | 2.126 | up | Peroxidase superfamily protein | Phenylpropanoid biosynthesis |
|  | BnaA10g25070D | AT5G05340 | 0.6358913 | 8.889739 | 3.873 | up | Peroxidase superfamily protein | Phenylpropanoid biosynthesis |
|  | BnaA01g01280D | AT4G36430 | 5.3854436 | 22.099816 | 2.105 | up | Peroxidase superfamily protein | Phenylpropanoid biosynthesis |
|  | BnaC09g37770D | AT5G19880 | 6.7325709 | 53.436058 | 3.056 | up | Peroxidase superfamily protein | Phenylpropanoid biosynthesis |
|  | BnaA02g04560D | AT5G19890 | 11.959647 | 51.096353 | 2.163 | up | Peroxidase superfamily protein | Phenylpropanoid biosynthesis |
|  | BnaA03g52510D | AT4G33420 | 13.280192 | 52.397868 | 2.048 | up | Peroxidase superfamily protein | Phenylpropanoid biosynthesis |
|  | BnaC09g37740D | AT5G19890 | 8.6635398 | 44.684582 | 2.434 | up | Peroxidase superfamily protein | Phenylpropanoid biosynthesis |
|  | BnaC02g04300D | AT5G12270 | 6.0970752 | 0.6442699 | -3.175 | down | 2-oxoglutarate (2OG) and Fe(II)-dependent oxygenase superfamily protein | Phenylpropanoid biosynthesis |
|  | BnaA01g21160D | AT5G12270 | 23.427803 | 4.2219978 | -2.405 | down | 2-oxoglutarate (2OG) and Fe(II)-dependent oxygenase superfamily protein | Phenylpropanoid biosynthesis |
|  | BnaC01g02300D | AT4G36430 | 21.463725 | 70.66399 | 1.787 | up | Peroxidase superfamily protein | Phenylpropanoid biosynthesis |
|  | BnaC09g50000D | AT5G05340 | 48.566196 | 160.79139 | 1.795 | up | Peroxidase superfamily protein | Phenylpropanoid biosynthesis |
|  | BnaC07g26270D | AT5G47910 | 21.111866 | 65.786729 | 1.707 | up | respiratory burst oxidase homologue D (RBOHD) | Plant-pathogen interaction |
|  | BnaA07g32970D | AT1G76650 | 11.105387 | 168.45924 | 3.991 | up | calmodulin-like 38 (CML38) | Plant-pathogen interaction |
|  | BnaC06g21690D | AT1G76650 | 1.1516698 | 64.529001 | 5.876 | up | calmodulin-like 38 (CML38) | Plant-pathogen interaction |
|  | BnaA06g30520D | AT5G47910 | 17.546431 | 62.970857 | 1.911 | up | respiratory burst oxidase homologue D (RBOHD) | Plant-pathogen interaction |
|  | BnaC06g37480D | AT1G76640 | 1.5242688 | 51.683332 | 5.151 | up | Calcium-binding EF-hand family protein | Plant-pathogen interaction |
|  | BnaCnng76780D | AT1G76650 | 0.8637523 | 12.394679 | 3.911 | up | calmodulin-like 38 (CML38) | Plant-pathogen interaction |
|  | BnaA09g00120D | AT4G01250 | 7.1931213 | 36.154531 | 2.397 | up | WRKY22 | Plant-pathogen interaction |
|  | BnaC05g13970D | AT1G18210 | 28.824094 | 124.04729 | 2.173 | up | Calcium-binding EF-hand family protein | Plant-pathogen interaction |
|  | BnaC06g37490D | AT1G76650 | 25.339916 | 198.10873 | 3.034 | up | calmodulin-like 38 (CML38) | Plant-pathogen interaction |
|  | BnaA09g08020D | AT5G66210 | 10.748918 | 45.660124 | 2.154 | up | calcium-dependent protein kinase 28 (CPK28) | Plant-pathogen interaction |
|  | BnaC03g32250D | AT3G01830 | 1.642346 | 19.275013 | 3.621 | up | Calcium-binding EF-hand family protein | Plant-pathogen interaction |
|  | BnaA07g12770D | AT5G66210 | 4.8707837 | 24.513622 | 2.399 | up | calcium-dependent protein kinase 28 (CPK28) | Plant-pathogen interaction |
|  | BnaAnng36110D | AT5G66210 | 4.226873 | 33.929721 | 3.073 | up | calcium-dependent protein kinase 28 (CPK28) | Plant-pathogen interaction |
|  | BnaA04g22040D | AT2G38470 | 10.42954 | 44.951059 | 2.175 | up | WRKY DNA-binding protein 33 (WRKY33) | Plant-pathogen interaction |
|  | BnaC07g27590D | AT2G38470 | 0 | 2.641942 | Inf | up | WRKY DNA-binding protein 33 (WRKY33) | Plant-pathogen interaction |
|  | BnaA03g17820D | AT2G38470 | 8.4188517 | 48.232981 | 2.586 | up | WRKY DNA-binding protein 33 (WRKY33) | Plant-pathogen interaction |
|  | BnaA03g27250D | AT3G01830 | 1.0948973 | 23.324386 | 4.481 | up | Calcium-binding EF-hand family protein | Plant-pathogen interaction |
|  | BnaCnng66020D | AT2G38470 | 19.841005 | 65.584925 | 1.793 | up | WRKY DNA-binding protein 33 (WRKY33) | Plant-pathogen interaction |
|  | BnaC03g45210D | AT2G27030 | 8.4828872 | 29.353497 | 1.859 | up | calmodulin 5 (CAM5) | Plant-pathogen interaction |
|  | BnaC08g17850D | AT1G18210 | 12.082827 | 46.905383 | 2.024 | up | Calcium-binding EF-hand family protein | Plant-pathogen interaction |
|  | BnaC09g08180D | AT5G66210 | 28.78112 | 89.243388 | 1.700 | up | calcium-dependent protein kinase 28 (CPK28) | Plant-pathogen interaction |
|  | BnaA06g38470D | AT1G18210 | 50.745448 | 217.54579 | 2.168 | up | Calcium-binding EF-hand family protein | Plant-pathogen interaction |
|  | BnaA05g34850D | AT2G38470 | 3.8978653 | 53.456521 | 3.845 | up | WRKY DNA-binding protein 33 (WRKY33) | Plant-pathogen interaction |
|  | BnaA07g32960D | AT1G76640 | 3.0485376 | 33.688874 | 3.534 | up | Calcium-binding EF-hand family protein | Plant-pathogen interaction |
|  | BnaC08g28520D | AT3G57530 | 8.1904794 | 28.102196 | 1.846 | up | calcium-dependent protein kinase 32 (CPK32) | Plant-pathogen interaction |
|  | BnaA09g53510D | AT5G42380 | 20.029716 | 149.06769 | 2.963 | up | calmodulin like 37 (CML37) | Plant-pathogen interaction |
|  | BnaC01g04530D | AT4G33720 | 118.60553 | 663.464 | 2.552 | up | CAP (Cysteine-rich secretory proteins, Antigen 5, and Pathogenesis-related 1 protein) superfamily protein | Plant-pathogen interaction |
|  | BnaC01g38680D | AT3G10300 | 0.2737243 | 31.909055 | 6.933 | up | Calcium-binding EF-hand family protein | Plant-pathogen interaction |
|  | BnaC04g06800D | AT2G38470 | 13.037771 | 63.263253 | 2.346 | up | WRKY DNA-binding protein 33 (WRKY33) | Plant-pathogen interaction |
|  | BnaC09g16820D | AT5G42380 | 37.258073 | 205.38768 | 2.530 | up | calmodulin like 37 (CML37) | Plant-pathogen interaction |
|  | BnaC07g16820D | AT5G66210 | 15.033246 | 80.413738 | 2.487 | up | calcium-dependent protein kinase 28 (CPK28) | Plant-pathogen interaction |
|  | BnaC03g21360D | AT2G38470 | 45.175477 | 164.65689 | 1.934 | up | WRKY DNA-binding protein 33 (WRKY33) | Plant-pathogen interaction |
|  | BnaA01g03260D | AT4G33720 | 141.70474 | 677.92142 | 2.326 | up | CAP (Cysteine-rich secretory proteins, Antigen 5, and Pathogenesis-related 1 protein) superfamily protein | Plant-pathogen interaction |
|  | BnaC09g02710D | AT5G49480 | 0 | 6.8572728 | Inf | up | Ca2+-binding protein 1 (CP1) | Plant-pathogen interaction |
|  | BnaA09g36820D | AT3G57530 | 11.952292 | 42.818155 | 1.909 | up | calcium-dependent protein kinase 32 (CPK32) | Plant-pathogen interaction |
|  | BnaA10g23840D | AT5G07390 | 0.1752823 | 1.9707251 | 3.559 | up | respiratory burst oxidase homolog A (RBOHA) | Plant-pathogen interaction |
|  | BnaC09g52610D | AT3G25600 | 23.833166 | 84.760865 | 1.898 | up | Calcium-binding EF-hand family protein | Plant-pathogen interaction |
|  | BnaA09g19500D | AT3G25600 | 38.229038 | 122.38504 | 1.746 | up | Calcium-binding EF-hand family protein | Plant-pathogen interaction |
|  | BnaC07g25470D | AT5G39670 | 71.705152 | 242.41729 | 1.825 | up | Calcium-binding EF-hand family protein | Plant-pathogen interaction |
|  | BnaCnng10300D | AT5G04170 | 2.4313028 | 10.90391 | 2.233 | up | Calcium-binding EF-hand family protein | Plant-pathogen interaction |
|  | BnaC03g03030D | AT5G07390 | 3.0503141 | 0.3752985 | -2.955 | down | respiratory burst oxidase homolog A (RBOHA) | Plant-pathogen interaction |
|  | BnaC02g01720D | AT5G07100 | 23.64522 | 5.6782326 | -1.990 | down | WRKY DNA-binding protein 26 (WRKY26) | Plant-pathogen interaction |
|  | BnaA03g47120D | AT4G25090 | 2.7031098 | 19.332501 | 2.906 | up | Riboflavin synthase-like superfamily protein | Plant-pathogen interaction |
|  | Gene ID | AGI number | Sck:fpkm | St:fpkm | log2FC(St/Sck) | Regulate | Gene annotation | Pathways |
| St Vs Sck | BnaA05g36800D | AT3G19710 | 4.036 | 0.215 | -4.466 | down | branched-chain aminotransferase4 (BCAT4) | Cysteine and methionine metabolism |
|  | BnaC08g36640D | AT2G19590 | 98.699 | 34.994 | -1.733 | down | ACC oxidase 1 (ACO1) | Cysteine and methionine metabolism |
|  | BnaA09g03700D | AT5G27381 | 119.138 | 38.333 | -1.874 | down | glutathione synthetase 2 (GSH3) | Cysteine and methionine metabolism |
|  | BnaA01g34610D | AT4G39950 | 233.083 | 56.759 | -2.275 | down | cytochrome P450, family 79, subfamily B, polypeptide 2 (CYP79B2) | Glucosinolate biosynthesis |
|  | BnaA04g06630D | AT4G13770 | 41.532 | 13.318 | -1.878 | down | cytochrome P450, family 83, subfamily A, polypeptide 1 (CYP83A1) | Glucosinolate biosynthesis |
|  | BnaA04g12790D | AT2G22330 | 65.476 | 19.720 | -1.969 | down | cytochrome P450, family 79, subfamily B, polypeptide 3 (CYP79B3) | Glucosinolate biosynthesis |
|  | BnaA06g11010D | AT1G16410 | 19.362 | 0.623 | -5.196 | down | cytochrome p450 79f1 (CYP79F1) | Glucosinolate biosynthesis |
|  | BnaA08g16100D | AT4G39950 | 36.154 | 3.445 | -3.629 | down | cytochrome P450, family 79, subfamily B, polypeptide 2 (CYP79B2) | Glucosinolate biosynthesis |
|  | BnaC01g00800D | AT4G39950 | 129.867 | 34.390 | -2.154 | down | cytochrome P450, family 79, subfamily B, polypeptide 2 (CYP79B2) | Glucosinolate biosynthesis |
|  | BnaC03g60820D | AT4G39950 | 24.345 | 2.546 | -3.495 | down | cytochrome P450, family 79, subfamily B, polypeptide 2 (CYP79B2) | Glucosinolate biosynthesis |
|  | BnaC05g12520D | AT1G16410 | 47.963 | 4.598 | -3.620 | down | cytochrome p450 79f1 (CYP79F1) | Glucosinolate biosynthesis |
|  | BnaC07g51280D | AT4G39950 | 136.255 | 43.315 | -1.891 | down | cytochrome P450, family 79, subfamily B, polypeptide 2 (CYP79B2) | Glucosinolate biosynthesis |
|  | BnaA01g20660D | AT3G49120 | 19.378 | 4.220 | -2.437 | down | peroxidase CB (PRXCB) | Phenylpropanoid biosynthesis |
|  | BnaA06g16150D | AT3G49120 | 68.564 | 15.694 | -2.365 | down | peroxidase CB (PRXCB) | Phenylpropanoid biosynthesis |
|  | BnaA09g53760D | AT4G08770 | 551.766 | 80.968 | -3.006 | down | Peroxidase superfamily protein | Phenylpropanoid biosynthesis |
|  | BnaC01g25860D | AT3G49120 | 7.105 | 0.888 | -3.237 | down | peroxidase CB (PRXCB) | Phenylpropanoid biosynthesis |
|  | BnaC08g20820D | AT3G49120 | 11.746 | 1.241 | -3.480 | down | peroxidase CB (PRXCB) | Phenylpropanoid biosynthesis |
|  | BnaCnng46860D | AT5G66390 | 114.522 | 41.935 | -1.687 | down | Peroxidase superfamily protein | Phenylpropanoid biosynthesis |
|  | BnaA07g12770D | AT5G66210 | 24.741 | 4.871 | -2.582 | down | calcium-dependent protein kinase 28 (CDPK28) | Plant-pathogen interaction |
|  | BnaA03g38480D | AT2G13790 | 16.691 | 3.774 | -2.382 | down | somatic embryogenesis receptor-like kinase 4 (SERK4) | Plant-pathogen interaction |
|  | BnaA09g36820D | AT3G57530 | 53.343 | 11.952 | -2.396 | down | calcium-dependent protein kinase 32 (CDPK32) | Plant-pathogen interaction |
|  | BnaA03g52720D | AT4G33720 | 144.281 | 0.000 | -Inf | down | CAP (Cysteine-rich secretory proteins, Antigen 5, and Pathogenesis-related 1 protein) superfamily protein | Plant-pathogen interaction |
|  | BnaA04g22040D | AT2G38470 | 63.996 | 10.430 | -2.855 | down | WRKY DNA-binding protein 33 (WRKY33) | Plant-pathogen interaction |
|  | BnaA06g30520D | AT5G47910 | 111.008 | 17.546 | -2.899 | down | respiratory burst oxidase homologue D (RBOHD) | Plant-pathogen interaction |
|  | BnaAnng07990D | AT5G47910 | 21.741 | 7.897 | -1.698 | down | respiratory burst oxidase homologue D (RBOHD) | Plant-pathogen interaction |
|  | BnaC02g38300D | AT5G47910 | 64.305 | 18.887 | -2.005 | down | respiratory burst oxidase homologue D (RBOHD) | Plant-pathogen interaction |
|  | BnaC03g45290D | AT2G13790 | 25.489 | 7.973 | -1.914 | down | somatic embryogenesis receptor-like kinase 4 (SERK4) | Plant-pathogen interaction |
|  | BnaC07g26270D | AT5G47910 | 133.270 | 21.112 | -2.896 | down | respiratory burst oxidase homologue D (RBOHD) | Plant-pathogen interaction |
|  | BnaA08g23120D | AT1G17420 | 0.901 | 0.000 | -Inf | down | lipoxygenase 3 (LOX3) | alpha-Linolenic acid metabolism |
|  | BnaA02g16020D | AT1G72520 | 27.795 | 0.366 | -6.484 | down | PLAT/LH2 domain-containing lipoxygenase family protein | alpha-Linolenic acid metabolism |
|  | BnaA07g33310D | AT1G77120 | 426.231 | 78.147 | -2.685 | down | alcohol dehydrogenase 1 (ADH1) | alpha-Linolenic acid metabolism |
|  | BnaC02g21440D | AT1G72520 | 56.998 | 0.853 | -6.300 | down | PLAT/LH2 domain-containing lipoxygenase family protein | alpha-Linolenic acid metabolism |
|  | BnaC06g37860D | AT1G77120 | 347.436 | 55.371 | -2.887 | down | alcohol dehydrogenase 1 (ADH1) | alpha-Linolenic acid metabolism |
|  | BnaC06g18370D | AT2G47001 | 35.067 | 2.512 | -4.041 | down | ATP binding cassette subfamily B4 (ABCB5) | ABC transporters |
|  | BnaA07g19210D | AT2G47001 | 68.231 | 4.721 | -4.091 | down | ATP binding cassette subfamily B4 (ABCB5) | ABC transporters |
|  | BnaA09g17560D | AT5G45340 | 86.653 | 5.327 | -4.261 | down | cytochrome P450, family 707, subfamily A, polypeptide 3 (CYP707A3) | Carotenoid biosynthesis |
|  | BnaC02g31560D | AT5G45340 | 200.633 | 18.926 | -3.644 | down | cytochrome P450, family 707, subfamily A, polypeptide 3 (CYP707A3) | Carotenoid biosynthesis |
|  | BnaC07g18360D | AT5G45340 | 13.440 | 2.201 | -2.848 | down | cytochrome P450, family 707, subfamily A, polypeptide 3 (CYP707A3) | Carotenoid biosynthesis |
|  | BnaC09g18860D | AT5G45340 | 94.721 | 10.265 | -3.443 | down | cytochrome P450, family 707, subfamily A, polypeptide 3 (CYP707A3) | Carotenoid biosynthesis |
|  | BnaA06g18440D | AT3G45640 | 201.780 | 41.972 | -2.503 | down | mitogen-activated protein kinase 3 (MPK3) | MAPK signaling pathway - plant |
|  | BnaC03g55440D | AT3G45640 | 207.859 | 51.197 | -2.259 | down | mitogen-activated protein kinase 3 (MPK3) | MAPK signaling pathway - plant |
|  | BnaC05g04210D | AT1G06160 | 2.916 | 0.000 | -Inf | down | octadecanoid-responsive Arabidopsis AP2/ERF 59 (ORA59) | Plant hormone signal transduction |
|  | BnaA02g25110D | AT5G47220 | 69.050 | 15.480 | -2.395 | down | ethylene responsive element binding factor 2 (ERF2) | Plant hormone signal transduction |
|  | BnaA06g35500D | AT5G47220 | 38.084 | 11.464 | -1.970 | down | ethylene responsive element binding factor 2 (ERF2) | Plant hormone signal transduction |
|  | BnaA08g20970D | AT1G22070 | 8.079 | 2.031 | -2.229 | down | TGA1A-related gene 3 (TGA3) | Plant hormone signal transduction |
|  | BnaC07g20040D | AT5G47220 | 43.930 | 5.229 | -3.308 | down | ethylene responsive element binding factor 2 (ERF2) | Plant hormone signal transduction |
|  | BnaC07g50210D | AT4G18710 | 29.875 | 10.503 | -1.746 | down | BRASSINOSTEROID-INSENSITIVE 2 (BIN2) | Plant hormone signal transduction |
|  | BnaC09g20090D | AT5G47220 | 45.861 | 13.978 | -1.952 | down | ethylene responsive element binding factor 2 (ERF2) | Plant hormone signal transduction |
|  | BnaCnng60520D | AT5G47220 | 170.832 | 51.023 | -1.981 | down | ethylene responsive element binding factor 2 (ERF2) | Plant hormone signal transduction |
|  | BnaA09g00120D | AT4G01250 | 52.422 | 7.193 | -3.103 | down | WRKY22 | Plant-pathogen interaction |
|  | BnaC08g19310D | AT1G20510 | 26.661 | 7.858 | -2.000 | down | OPC-8:0 CoA ligase1 (OPCL1) | alpha-Linolenic acid metabolism |
|  | BnaA08g21760D | AT1G20510 | 40.716 | 8.279 | -2.536 | down | OPC-8:0 CoA ligase1 (OPCL1) | alpha-Linolenic acid metabolism |
|  | BnaA02g15990D | AT1G72450 | 25.302 | 3.405 | -3.131 | down | jasmonate-zim-domain protein 6 (JAZ6) | Plant hormone signal transduction |
|  | Gene ID | AGI number | Rck:fpkm | Rt:fpkm | log2FC(Rt/Rck) | Regulate | Gene annotation | Pathways |
| Rt Vs Rck | BnaA03g02840D | AT5G10240 | 10.714 | 24.386 | 1.123 | up | asparagine synthetase 3 (ASN3) | Alanine, aspartate and glutamate metabolism |
|  | BnaA03g34780D | AT3G17820 | 0.908 | 4.846 | 2.352 | up | glutamine synthetase 1.3 (GLN1.3) | Alanine, aspartate and glutamate metabolism |
|  | BnaC03g04190D | AT5G10240 | 8.256 | 20.981 | 1.282 | up | asparagine synthetase 3 (ASN3) | Alanine, aspartate and glutamate metabolism |
|  | BnaA08g26000D | AT1G10070 | 27.162 | 5.172 | -2.456 | down | branched-chain amino acid transaminase 2 (BCAT-2) | Cysteine and methionine metabolism |
|  | BnaAnng32880D | AT3G57050 | 4.716 | 1.553 | -1.666 | down | cystathionine beta-lyase (CBL) | Cysteine and methionine metabolism |
|  | BnaC08g36640D | AT2G19590 | 45.751 | 142.514 | 1.576 | up | ACC oxidase 1 (ACO1) | Cysteine and methionine metabolism |
|  | BnaC09g20710D | AT2G02390 | 12.893 | 5.155 | -1.386 | down | glutathione S-transferase zeta 1 (GSTZ1) | Tyrosine metabolism |
|  | BnaA01g06830D | AT5G67400 | 8.375 | 21.231 | 1.279 | up | RHS19, ROOT HAIR SPECIFIC 19 hydrogen peroxide catabolic process, oxidation-reduction process, response to oxidative stress | Phenylpropanoid biosynthesis |
|  | BnaA05g04030D | AT2G44460 | 2.250 | 6.113 | 1.379 | up | beta glucosidase 28 (BGLU28) | Phenylpropanoid biosynthesis |
|  | BnaA05g29140D | AT3G09260 | 1.591 | 17.305 | 3.380 | up | PYK10 | Phenylpropanoid biosynthesis |
|  | BnaA09g05060D | AT5G24070 | 0.000 | 1.950 | Inf | up | Peroxidase superfamily protein | Phenylpropanoid biosynthesis |
|  | BnaC05g43610D | AT3G09260 | 7.956 | 66.504 | 3.000 | up | PYK10 | Phenylpropanoid biosynthesis |
|  | BnaA02g02640D | AT5G15490 | 3.328 | 10.576 | 1.605 | up | Encodes one of four UDP-glucose dehydrogenase UGD) genes. | Pentose and glucuronate interconversions |
|  | BnaA01g13540D | AT4G23920 | 19.458 | 7.777 | -1.387 | down | UDP-D-glucose/UDP-D-galactose 4-epimerase 2 (UGE2) | Amino sugar and nucleotide sugar metabolism |
|  | BnaA03g52720D | AT4G33720 | 5.915 | 50.349 | 3.026 | up | CAP (Cysteine-rich secretory proteins, Antigen 5, and Pathogenesis-related 1 protein) superfamily protein | Plant-pathogen interaction |
|  | BnaA08g23120D | AT1G17420 | 3.544 | 1.275 | -1.538 | down | lipoxygenase 3 (LOX3) | alpha-Linolenic acid metabolism |
|  | BnaA03g38210D | AT2G06050 | 79.877 | 26.244 | -1.669 | down | oxophytodienoate-reductase 3 (OPR3) | alpha-Linolenic acid metabolism |
|  | BnaA07g33310D | AT1G77120 | 146.372 | 327.877 | 1.100 | up | alcohol dehydrogenase 1 (ADH1) | alpha-Linolenic acid metabolism |
|  | BnaC06g37860D | AT1G77120 | 187.929 | 438.945 | 1.160 | up | alcohol dehydrogenase 1 (ADH1) | alpha-Linolenic acid metabolism |
|  | BnaA01g30560D | AT3G12500 | 1.614 | 11.845 | 2.812 | up | basic chitinase (HCHIB) | MAPK signaling pathway - plant |
|  | BnaC08g19310D | AT1G20510 | 56.475 | 25.616 | -1.204 | down | OPC-8:0 CoA ligase1 (OPCL1) | alpha-Linolenic acid metabolism |
|  | BnaA08g21760D | AT1G20510 | 65.425 | 29.809 | -1.197 | down | OPC-8:0 CoA ligase1 (OPCL1) | alpha-Linolenic acid metabolism |
